# Benchmarking Clinical Speech Recognition and Information Extraction: New Data, Methods, and Evaluations

**DOI:** 10.2196/medinform.4321

**Published:** 2015-04-27

**Authors:** Hanna Suominen, Liyuan Zhou, Leif Hanlen, Gabriela Ferraro

**Affiliations:** ^1^Canberra Research LaboratoryMachine Learning Research GroupNICTACanberra, ACTAustralia; ^2^College of Engineering and Computer ScienceAustralian National UniversityCanberra, ACTAustralia; ^3^Faculty of HealthUniversity of CanberraCanberra, ACTAustralia; ^4^Department of Information TechnologyUniversity of TurkuTurkuFinland

**Keywords:** computer systems evaluation, data collection, information extraction, nursing records, patient handoff, records as topic, speech recognition software

## Abstract

**Background:**

Over a tenth of preventable adverse events in health care are caused by failures in information flow. These failures are tangible in clinical handover; regardless of good verbal handover, from two-thirds to all of this information is lost after 3-5 shifts if notes are taken by hand, or not at all. Speech recognition and information extraction provide a way to fill out a handover form for clinical proofing and sign-off.

**Objective:**

The objective of the study was to provide a recorded spoken handover, annotated verbatim transcriptions, and evaluations to support research in spoken and written natural language processing for filling out a clinical handover form. This dataset is based on synthetic patient profiles, thereby avoiding ethical and legal restrictions, while maintaining efficacy for research in speech-to-text conversion and information extraction, based on realistic clinical scenarios. We also introduce a Web app to demonstrate the system design and workflow.

**Methods:**

We experiment with Dragon Medical 11.0 for speech recognition and CRF++ for information extraction. To compute features for information extraction, we also apply CoreNLP, MetaMap, and Ontoserver. Our evaluation uses cross-validation techniques to measure processing correctness.

**Results:**

The data provided were a simulation of nursing handover, as recorded using a mobile device, built from simulated patient records and handover scripts, spoken by an Australian registered nurse. Speech recognition recognized 5276 of 7277 words in our 100 test documents correctly. We considered 50 mutually exclusive categories in information extraction and achieved the F1 (ie, the harmonic mean of Precision and Recall) of 0.86 in the category for irrelevant text and the macro-averaged F1 of 0.70 over the remaining 35 nonempty categories of the form in our 101 test documents.

**Conclusions:**

The significance of this study hinges on opening our data, together with the related performance benchmarks and some processing software, to the research and development community for studying clinical documentation and language-processing. The data are used in the CLEFeHealth 2015 evaluation laboratory for a shared task on speech recognition.

##  Introduction

### Information Flow Failures

Information flow, defined as channels, contact, communication, or links to pertinent people [1], is critical in health care. Failures in information flow lead to preventable adverse events, including delays in diagnosis and intervention, administration of incorrect treatments, and missed or duplicated tests among others [2-4]. In Australian hospitals, these failures are associated with over a tenth of preventable adverse events. Information flow is critical in clinical handover, when a clinician or group of clinicians is transferring professional responsibility and accountability, for example, at shift change [3].

Nursing handover is a form of clinical narrative [5], where the documented (written) material is only a small component of the complete information flow. There are multiple approaches to clinical handover at shift change; however, nursing handover typically occurs with a combination of whole-team in a private area, followed by whole-team in the presence of the patient or carer. Best practice in Australian hospital settings [6,7] recommends verbal handover in the patient’s presence, supplemented with written material.

### Australian Privacy Laws

The Australian National Health and Medical Research Council [8] places a number of restrictions on the use of Australian clinical data, most notably, avoidance of so-called *deidentified* data. The data referenced as *deidentified* in US publications [9,10] is considered as *reidentifiable* under Australian privacy law [8]. While approaches exist for semiautomatically deidentifying clinical texts [11,12], all such processes (whether automatic or manual) do not meet the stringent privacy requirements of Australian law.

An audio recording of a complete nursing handover requires ethical consenting of the nursing team, patients, visitors, and all other incidental clinical staff. It is difficult to obtain a “natural” recording—that could be provided without restriction on its use—under such conditions. Audio recordings also present significant difficulties in terms of identification of patients [13]. Reidentifiable data [8] must have restricted use, appropriate ethical use, and approval from all data generators (eg, patients, nurses, other clinicians’, and visitors at the wards).

Ethical deidentification of the nursing handover for open data is not realistic. The *British Medical Journal* recommends [14] not publishing verbatim responses or transcriptions of clinical discussions. Existing sources of clinical data have limitations such as research-only use [15], nondisclosure of data [16], or limited commercial licenses [17].

In the case of clinical nursing notes and handover, precise data does not exist in an open form. By open we mean without restriction [18], including commercial use. Due to the lack of existing datasets and the difficulty of providing an ethically sound “free” data resource, we have developed a synthetic dataset that closely matches the typical data found in a nursing shift change. Synthetic clinical documents have also been used in other clinical informatics studies. For example in 2013-2014, the *MedNLP* track on medical natural language processing (NLP) used synthetic clinical notes [19].

Free-form text, as an entry type, is essential to release clinicians’ time from documentation for other tasks [20-22]. NLP (a.k.a. automated text analysis or text mining) [10,23-28], including speech recognition (SR) and information extraction (IE), provides a way to fill out a handover form for clinical proofing and sign-off (see Multimedia Appendix 1), but this cascaded system evokes significant research challenges.

The development of these techniques is hindered by access to data for research, development, and evaluation [29]. Medical shared tasks by, for example, *NII Testbeds and Community for Information access Research* [19], *Text Retrieval Conference* [30], and *Conference and Labs of the Evaluation Forum* (CLEF) *eHealth* [31] (see this reference also for a review of related shared tasks), have provided deidentified datasets to researchers, who developed new clinical language technologies to improve clinical notes and credit patient outcomes. In 2013, the *Health Design Challenge* had a shared task aiming to make clinical documents more usable by and meaningful to patients, their families, and others who take care of them [32]. This design/visualization task attracted over 230 teams to participate.

By providing an open clinical dataset, that includes verbatim conversations and associated audio recordings, we anticipate a greater impact from the shared computational tasks, and increased development in natural language technologies for clinical text. Consequently, the significance of this study hinges not only on opening our data and some processing software to the research and development community, but also on publishing our performance evaluation results as a benchmark for tracking of performance improvements in time.

## Methods

### Data Creation

#### Creation Process

**Figure 1:**
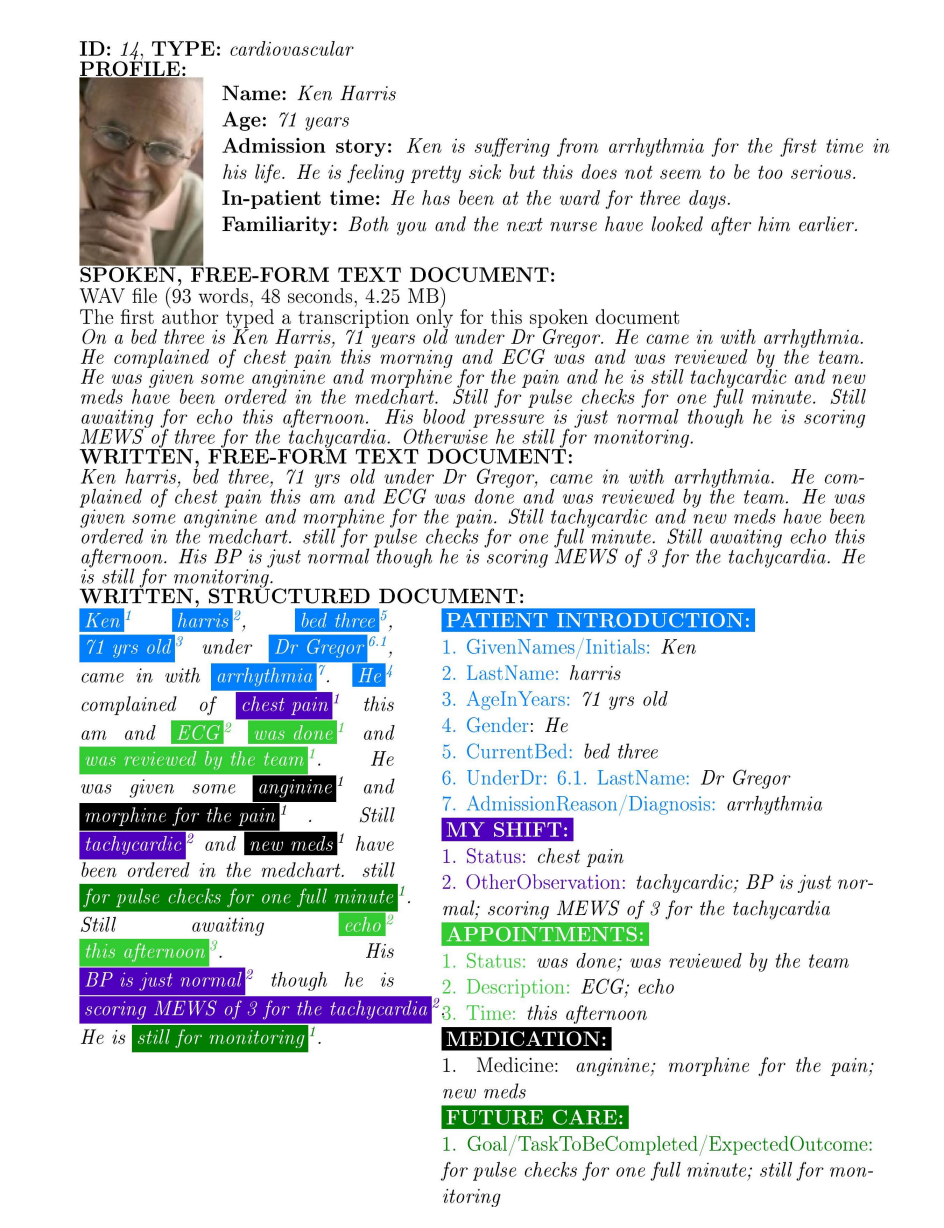
An example record that originates from our dataset.

We created a synthetic dataset of 101 handover records (see Multimedia Appendix 1). Each record consisted of a patient profile; spoken, free-form text document; written, free-form text document; and written, structured document (Figure 1 shows this). The creation process included the following five steps: (1) generation of patient profiles; (2) creating a synthetic, but realistic nursing handover dataset, in collaboration with a registered nurse (RN); (3) development of a structured handover form; (4) using this form and the written, free-form text documents to create written, structured documents; and (5) creation of spoken, free-form text documents.

#### Generation of Patient Profiles

The patient profile was developed using common user profile generation techniques [33]. The first author of this paper (Adj/Prof in machine learning for communication and health computing) considered an imaginary medical ward in Australia. With an aim for balance in patient types, she created simulated profiles for 101 patients. This included 1 sample patient together with 25 cardiovascular, 25 neurological, 25 renal, and 25 respiratory patients of the ward. These patient types were chosen because they represent the most common chronic diseases and national health priority areas in Australia [34]. This provided a balanced demographic sample from which various handover scenarios could be created.

Each imaginary profile was given a stock photo from a royalty-free gallery, name, age, admission story, in-patient time, and familiarity to the nurses giving and receiving the handover. All patients were adults, but both young and old people were included. Some patients were recently admitted to the ward, some had been there for some days already, and some were almost ready to be discharged. For some patients, the in-patient time was short and for other patients it was longer. Within the admission story, the reason for admission was always an acute condition, but some patients had also chronic diseases.

#### Creation of Written, Free-Form Text Documents

The first author created a synthetic, written, free-form text document for the sample profile and supervised a RN in creating these documents for the remaining 100 profiles.

The RN had over twelve years experience from clinical nursing. She spoke Australian English as her second language and was originally from the Philippines. The RN’s written consent was obtained for gathering, using, and releasing the spoken and written documents she created. She performed all these creative speaking and writing tasks as a National Information and Communications Technology, Australia (NICTA) employee alone in an office environment.

The RN was guided to imagine herself working in the medical ward and delivering verbal handovers to another nurse at a nursing shift change by the patient’s bedside (see Multimedia Appendix 1). The handovers were to be monologues that include all handover information at once rather than discussions.

The RN was asked to write, for each patient profile, a realistic, but fully imaginary text document (ie, TXT file) as if she was talking and using normal wordings. The document length was set to 100-300 words.

#### Development of a Structured Handover Form

**Figure 2:**
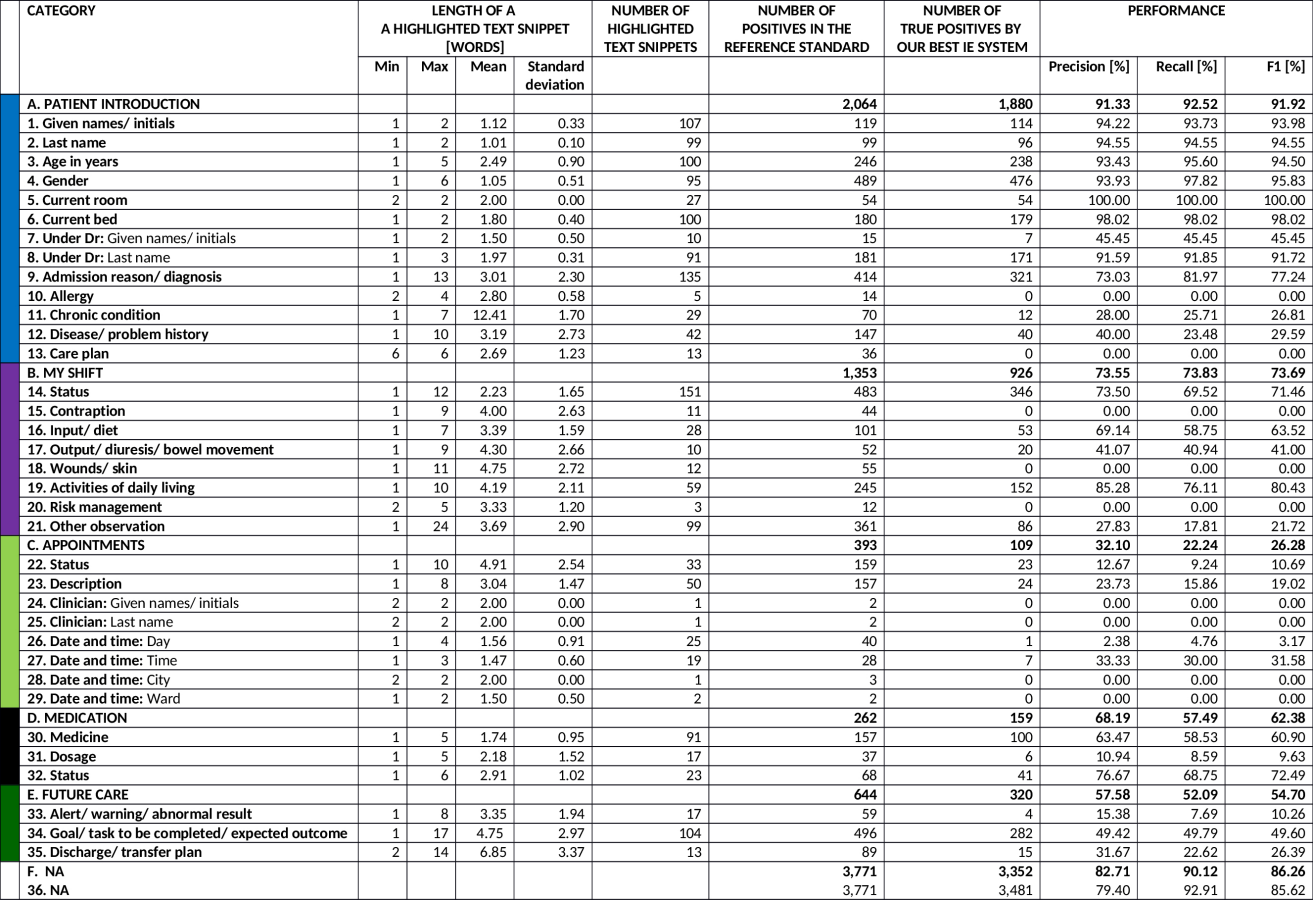
Descriptive statistics of text snippets highlighted by the registered nurse in the 101 written, structured documents used as a reference standard in information extraction together with the performance of our best information extraction system. RN: registered nurse; RS: reference standard; IE: information extraction; NA: not applicable; min: minimum; max: maximum.

In consultation with Nursing Handover domain experts, the first and third authors developed a handover form (Figure 2 shows this) to be filled out. The form is compatible with existing handover forms, and matches the Australian and international standards/best practice for handover communication [35,36], as well as mimicks the RN’s practical experiences from two Australian states/territories.

The form consisted of six headings (ie, *HANDOVER NURSE*, *PATIENT INTRODUCTION*, *MY SHIFT*, *APPOINTMENTS*, *MEDICATION*, and *FUTURE CARE*) with mutually exclusive categories (ie, *Title*, *Given names/initials*, *Last name*, and other subheadings together with subsubheadings like *Year*, *Month*, *Day* under *Date of birth*) for patient information, supplemented with the category of *Not Applicable* (*NA*) for irrelevant information. The number of categories was in total fifty with five, eighteen, eight, twelve, three, and three categories under *HANDOVER NURSE*, *PATIENT INTRODUCTION*, *MY SHIFT*, *APPOINTMENTS*, *MEDICATION*, and *FUTURE CARE*, respectively, and one category for *NA*.

This form structure is also consistent with the five-step nursing process model by the American Nurses Association (ANA): Assessment, Diagnosis, Outcomes/Planning, Implementation, and Evaluation [37].

ANA specifies that information about the first three steps should be documented under the patient’s care plan in the patient’s record so that nurses and other health care professionals caring for the patient have access to it. The Assessment step refers to a nurse collecting and analyzing patient information, including, physiological data together with psychological, sociocultural, spiritual, economic, and life-style factors. The Diagnosis step refers to his/her clinical judgment about the patient’s response to actual or potential health conditions or needs. The Outcomes/Planning step refers to the nurse setting, based on the two previous steps, measurable and achievable short- and long-range goals for this patient. In our form, these three steps were covered under the headings of *PATIENT INTRODUCTION* with own, specific subheadings of *Admission reason/diagnosis* and *Care plan* for the initial Diagnosis and Outcomes/Planning steps.

The Implementation step refers to the implementation of nursing care in accordance with the care plan in order to assure the continuity of care for the patient during hospitalization and in preparation for discharge. Also, this delivered care is to be documented in the patient’s record. In our form, it was covered under the headings of *MY SHIFT*, *MEDICATION*, *APPOINTMENTS*, and *FUTURE CARE*.

The Evaluation step refers to the continuous evaluation of the patient’s status and the effectiveness of the nursing care and the respective modifications of the (written) care plan. Our form captured this step by considering the longitudinal series of handover documents in time.

#### Creation of Written, Structured Documents

The first author created a model structuring of the sample patient’s written, free-form text document with respect to the mutually exclusive categories of the handover form and supervised the RN in creating these written, structured documents for the remaining 100 profiles. The RN proofed and agreed on this sample structuring. The first author installed Protégé 3.1.1 with the Knowtator 1.9 beta [38] on the RN’s computer and guided her in using it to structure the documents (see Multimedia Appendix 1).

The RN was reminded that, on one hand, not all documents include information for all form categories and, on the other hand, some documents have relevant information to a given category multiple times (eg, if a given patient was referred to in a document with both a given name Michael and nickname Mike, both these occurrences were to be assigned to the category of *PATIENT INTRODUCTION: Given names/initials*).

The first and second author performed light proofing of these 101 structured documents in total. More precisely, they improved the consistency in including/excluding articles or titles, as well as in marking gender information in each document if it was available.

#### Creation of Spoken, Free-Form Documents

The first author supervised the RN in creating the spoken, free-form text documents by reading the 100 written free-form text documents out loud as the nurse giving the handover. She was guided to try to speak as naturally as possible, avoid sounding like reading text, and repeat the take until she was satisfied with the outcome (see Multimedia Appendix 1).

The Olympus WS-760M digital recorder and Olympus ME52W noise-canceling lapel-microphone (see Multimedia Appendix 1) that were previously used and shown to produce a superior word correctness in SR [36] captured the RN’s voice. The use of the recorder and microphone was practiced before the actual recording and the recording took place in a quiet office environment.

The first author edited each Windows Media Audio (WMA) audio recording to include only one handover document. This included assuring the file beginning and end did not include recordings that the RN was unsatisfied with, file identifiers, or other additional content.

### Processing and Evaluation Methods for Speech Recognition

#### Processing Methods

We used Dragon Medical 11.0 to convert the audio files to written, free-form text documents. This software was initialized with respect to the RN’s details of age of 22-54 years and accent of Australian English, and trained to her voice by recording her reading the document of The Final Odyssey (3893 words, 29 minutes 22 seconds, 4 minutes needed) using the aforementioned recorder and microphone. This training, tailoring, or adaptation to a speaker’s voice was left minimal, since it could limit comparability with other studies and might not be feasible for every clinician in practice. To meet the software requirements, the first author converted WMA recordings from stereo to mono tracks and exported them from WMA to WAVeform (WAV) files on Audacity 2.0.3 [39].

We compared the Dragon vocabularies of *general*, *medical*, *nursing*, *cardiology*, *neurology*, and *pulmonary disease*. That is, we used the most general clinical vocabulary of *general*, the vocabulary suitable for a medical ward (ie, *medical*), the vocabulary suitable for nursing handovers (ie, *nursing*), and the vocabularies that were the closest matches with our patient types (ie, *cardiology* for cardiovascular patients, *neurology* for neurological patients, and *pulmonary disease* for respiratory patients).

#### Evaluation Methods

We applied the SCLITE scoring tool of the SR Scoring Toolkit 2.4.0 [40] in the analysis of the correctly recognized, substituted, inserted, and deleted words. The reference standard (RS) in all comparisons consisted of the original written, free-form text documents by the RN (ie, not transcriptions by hand), where punctuation was removed and capitalization was not considered as a distinguishing feature.

We chose the vocabulary resulting in the best performance in terms of the correctly recognized words (see the Results section) for a more detailed error analysis. The correct, substituted, inserted, and deleted words were defined by the aforementioned SCLITE scoring tool. As the most fundamental concept in this analysis, we measured the phonetic similarity (PS), defined as a perceptual distance between speech sounds [41], between words in the RS and speech-recognized text in order to find sound-alike substitution errors (eg, “four” vs “for” or “doctors signed” vs “dr san”) for their correction. In the error analysis, we used the entire dataset and the subset that affects the IE system (ie, “inside” refers to text identified as relevant to the slots of the handover form). The correction could be based on linguistic postprocessing that combines PS with grammatical context [42-44].

We implemented a simple PS measure, which combines the Double Metaphone phonetic encoding algorithm [45,46] on the Apache Commons Metaphone [47] with the unweighted edit distance of the SimMetrics library [48]. We chose this algorithm because it approximates accented English from Slavic, Germanic, French, and Spanish, among others languages, and can be therefore seen as suitable for our accented RN’s speech. The encoding algorithm translated each consonant into a limited set of characters where similar sounds are represented by the same character (eg, “b” and “p” both sound like “p”). The unweighted edit distance calculated the similarity between the encoded words or word sequences as the minimum number of substitution, insertion, and deletion operations required to transform an encoded word into another. Because the algorithm is designed to encode a single word at a time, we first encoded each word in a multi-word sequence, then combined the encoded words as a sequence, and finally calculated the edit distance to measure the similarity between the sequences.

### Processing and Evaluation Methods for Information Extraction

#### Processing Methods

We used our expert-annotated dataset to train and evaluate IE systems. We considered this learning problem as a task where each word in text is considered as an entity with features and the goal is to assign it automatically to one or none of the categories. We chose to apply the conditional random field (CRF) [49], a probabilistic model for processing, segmenting, and labeling sequence data. This method solved the IE task by assigning precisely one category to each word of the document(s) based on patterns it has learned by observing words and the RN’s expert-annotated categories, as well as the enriched feature representation of the words and their context. We adopted an open-source implementation of CRFs called CRF++ [50]*.*

We generated the features by processing the original records using Stanford CoreNLP (English grammar) by the *Stanford Natural Language Processing Group* [51], MetaMap 2012 by the *US National Library of Medicine* [52], and Ontoserver by the *Australian Commonwealth Scientific and Industrial Research Organisation* [53] (Tables 1-3). Our best system used eight syntactic, three semantic, and twelve statistical feature types. We also experimented with additional feature types, but this did not contribute to the IE system performance.

In the CRF++ template, we defined in the *unigram part* that we use all features of the current location alone; all features of the previous location alone; all features of the next location alone; the pairwise correlations of the previous and current location over all features; the pairwise correlations of the current and next location over all features; and the combination of all features in the current location. In the *binary part*, we combined the predicted category for the previous location and the features of the current location to form a new feature.

**Table 1:** Experimented syntactic features.

ID	Name	Definition	Example	Software	In our best IE system
1	Word	Word itself	“Patients” or “had”	None	Yes
2	Lemma	Lemma of the word	“patients” or “have”	CoreNLP	Yes
3	NER^a^	NER^a^ tag of the word for named entities (ie, person, location, organization, other proper name) and numerical entities (ie, date, time, money, number)	“number” for “5”	CoreNLP	Yes
4	POS^b^	POS^b^ tag of the word	“IN” (ie, preposition) for “in”, “NN” (ie, common noun as opposed to Proper Name, “PN”) for “bed”, “CN” (ie, cardinal number) for “5”	CoreNLP	Yes
5	Parse tree	Parse tree of the sentence from the root to the current word	“ROOT-NP-NN” (ie, root-noun phrase-common noun) for “5” in “In bed 5 we have...”	CoreNLP	Yes
6	Basic dependents	Basic dependents of the word	“Cardinal number 5” that refers to the bed ID for “bed” in “In bed 5 we have...”	CoreNLP	Yes
7	Basic governors	Basic governors of the word	Preposition “in” and subject “we” for “have” in “In bed 5 we have...”	CoreNLP	Yes
8	Phrase	Phrase that contains this word	“In bed 5” for “bed” in “In bed 5 we have”...	MetaMap	Yes

^a^ NER = named entity recognition

^b^ POS = part of speech

**Table 2:** Experimented semantic features.

ID	Name	Definition	Example	Software	In our best IE system
9	Top 5 candidates	Top 5 candidates retrieved from UMLS^a^	“BP” may refer to, for example, “Bachelor of Pharmacy”**,** “bedpan”, “before present”, “birthplace”, or ”blood pressure”	MetaMap	Yes
10	Top mapping	Top UMLS^a^ mapping for the concept that is the best match with a given text snippet	“pneumonia” is a type “respiratory tract infection”	MetaMap	Yes
11	Medication score	1 if the word is a full term in ATCL^b^; else 0.5 if it can be found in ATCL^b^; 0 otherwise	1 for “acetylsalicylic acid”	NICTA	Yes

^a^ UMLS = Unified Medical Language System

^b^ ATCL = Anatomical Therapeutic Chemical List

**Table 3:** Experimented feature types, statistical features.

ID	Name	Definition	Example	Software	In our best IE system
12	Location	Location of the word on a ten-point scale from the beginning of the document to its end	“1” for the first word and “10” for the last word	NICTA	Yes
13	Normalized term frequency	Number of times a given term occurs in a document divided by the maximum of this term frequency over all terms in the document		NICTA	No
14	Top 5 candidates’	As 9 using SNOMED-CT-AU^a^		Ontoserver	No
15	Top mapping’	As 10 using SNOMED-CT-AU^a^		Ontoserver	No
16	Top 5 candidates’’	As 9 using AMT^b^		Ontoserver	No
17	Tom mapping’’	As 10 using AMT^b^		Ontoserver	No

^a^ SNOMED-CT-AU = Systematized Nomenclature of Medicine - Clinical Terms - Australian Release

^b^ AMT = Australian Medicines Terminology

#### Evaluation Methods

To evaluate the system performance, we used cross-validation (CV) with 100 documents for training and leaving out one for testing (ie, *leave-one-out,* LOO, CV over 101 documents). In addition, to assess the task difficulty and adequacy of the amount of data used for training, we computed system learning curves for training set sizes of 20, 40, 60, and 80 documents (together with the aforementioned training with 100 documents). For this purpose, we chose 21 documents to be used for testing by sampling the entire document set randomly without replacement. Then, we chose the documents to be used for training by sampling the remaining set of documents randomly without replacement. That is, we used all remaining documents for training when the training set size was 80, and otherwise chose a document subset of an appropriate size randomly without replacement. In order to assess the contribution of each feature to the overall system performance, we performed a leave-feature-out experiment on our best system and LOO CV. See, for example, [54] for these evaluation methods.

In these evaluations, we measured the Precision, Recall, and F1 (ie, the harmonic mean of Precision and Recall) as implemented in use in *CoNLL 2000 Shared Task on Chunking* [55]. We evaluated performance both separately in every category and over all categories. When evaluating the latter performance, we used both macro- and micro-averaging over all other categories than *NA*. We also documented the performance in the dominating category of *NA* category-specifically. Because our desire was to perform well in all classes, and not only in the majority classes, the macro-averaged results are to be emphasized over the micro-averaged results.

We also used two baseline systems: (1) the random baseline assigned a class to each word randomly and (2) the majority baseline the most frequent class (ie, *Future goal/Task to be completed/Expected outcome*).

Finally, to assess the stability and robustness of our categorization form, expert annotations, and IE system, we performed an experiment, where our goal was to predict only the highest-level classification task to the heading categories of *HANDOVER NURSE*, *PATIENT INTRODUCTION*, *MY SHIFT*, *APPOINTMENTS*, *MEDICATION, FUTURE CARE*, and *NA*. We compared two systems with exactly the same features, template, and LOO CV setting. The first system was trained on subheading and subsubheading level annotations as above and then its predictions were abstracted to the highest level. The second system was trained on these heading-level categories directly.

This experiment tested the null hypothesis of detailed annotations not being helpful for system performance. On the one hand, if we gained evidence to support the alternative hypothesis of detailed annotations being helpful, we would need to divide the more loosely defined and verbose categories (eg, *Care plan* and *Future goal/Task to be completed/Expected outcome*) to subcategories. On the other hand, if we accepted the null hypothesis, we could be satisfied with our form structure and annotations. This division of headings to subheadings would also then be a likely cure for issues we observed in our former study [36] that used a handover form with five high-level headings only.

In any case, even though it was more laborious to annotate free-form text with respect to the fifty categories of our form versus using the seven heading-level categories only, automatically generated structured documents, enabled by these more detailed annotations have many benefits. Namely, they support the documents reuse in computerized decision making and surveillance in health care better than the loosely classified documents.

## Results

### National Information and Communications Technology, Australia Synthetic Nursing Handover Data, Descriptive Statistics and Validation

The released dataset, called NICTA Synthetic Nursing Handover Data [56], included the following data records: (1) Dragon initialization details for the RN (ie, 1. DOCX for the written, free-form text document that originates from the Dragon software release and is to be used as the RS text and 2. WMA for the spoken, free-form text document by the RN) in the folder *handoverdata/initialisation* of the expanded file *handoverdata.ZIP*; (2) 100 patient profiles (DOCX) created by the first author and the respective 100 written, free-form text documents (TXT) created by the RN together with the sample text by the first author in the folders *handoverdata/ 100profiles* and *handoverdata/101writtenfreetextreports*, respectively; (3) 100 spoken, free-form text documents by the RN (WAV) in the folder *handoverdata/ 100audiofiles*; (4) 100 speech-recognized, written, free-form text documents for each of the six vocabularies (TXT) in the vocabulary-specific subfolders (eg, *Dragon-cardiology*) of the folder *handoverdata/100x6speechrecognised*; and (5) 101 written, structured documents for IE that include the RS text, features used by our best system, and form categories with respect to the RS and our best IE system when using LOO CV and the respective template (TXT, CRF++ format) in the folder *handoverdata/101informationextraction*.

Descriptive statistics of the dataset are given in Tables 4 and 5 and Figure 2.

### Data Release

The licensing constraints were set as follows, the license of the spoken, free-form text documents (ie, WMA and WAV files) was set as “Creative Commons - Attribution Alone - Noncommercial - No Derivative Works” [57], for the purposes of testing SR and language processing algorithms in order to allow others to test their computational methods against these files with appropriate acknowledgment. The license of the remaining documents (ie, DOCX and TXT files) was set as “Creative Commons-Attribution Alone” [58] with our intention to allow others to use these text and image files for any purpose with appropriate acknowledgment. In both cases, the acknowledgment requirement is to cite this paper.

All documents were made publicly available on the Internet. They will be used in the CLEFeHealth 2015 evaluation laboratory for a shared task on SR [59].

### Data Validation

The technical pipeline (ie, recorded voice, transcription, analysis) has been validated in clinical settings and published [36,60,61]. We have also evaluated the model of the handover [60,61] and systematically reviewed relevant technical literature [62].

Although the data we provided are a simulation of nursing handover, the written text for the handover scenario was based upon 150 live audio recordings of nursing handover in several Sydney-based hospitals [36,60,61]. These recordings were manually transcribed under confidentiality conditions and the results used to inspire new handover scenarios. The audio recordings contained 71/150 examples (47.3%) with a single person speaking, 59/150 (39.3%) with two people speaking, and 20/150 (13.3%) with three people speaking. Based on these recordings, and anecdotal evidence from clinical experts, a single speaker scenario appears to occur in half of the team handovers in the Australian Capital Territory and New South Wales-based hospitals. (Each state, and in some cases each health jurisdiction, in Australia has a slightly different model for handover. Discussions with domain experts suggested similar percentages in all health jurisdictions, but we are not aware of any systematic evidence.) Our clinical advisers noted that English-as-a-second-language is common in nursing handover. Patient voices were present only in 2 of the 150 recordings. The final scenarios, including audio files and transcripts, were presented to Nursing Managers and verified as a reasonable facsimile of true handover scenarios.

Finally, also the technical performance, including the suitability of different vocabularies for SR and features resulting in the best IE system, was similar [36] and in this current study. When using the same SR software with the nursing vocabulary and very similar approach for recording and initialization, the recognition correctness was from 0.62 (accented female) through 0.64 (native female) to 0.71 (native male) in [36]. Now, this correctness was 0.73, as we will learn in the next subsection. Similarly in IE, the F1 was 0.62 in both cases when macro-averaging over the five form-categories. For the irrelevant text, F1 was 0.85 in the former study and 0.86 now. These IE experiments used CRF++ with very similar features, template setting, and form headings.

**Table 4:** Descriptive statistics of the 100 written, free-form text documents produced by the RN.

Descriptor	Subdescriptor	Patient type	Patient type	Patient type	Patient type	All
		Cardiovascular	Neurological	Renal	Respiratory	
Documents	Number documents	25	25	25	25	100
	Number of words	1795	1545	1818	2119	7277
	Number of unique words	556	500	496	604	1304
	Number of inside words	1140	1006	1086	1305	4547
	Number of unique inside words	447	397	408	483	1106
Number of words in a document	Minimum	19	26	29	31	19
	Maximum	162	106	149	209	209
	Mean	70	60	71	83	71
	SD	37	22	33	39	34
Top 10 words in documents	1^st^ (n)^a^	and (95)	and (64)	and (88)	and (100)	and (347)
	2^nd^ (n)^a^	he (59)	is (60)	is (72)	is (69)	is (256)
	3^rd^ (n)^a^	for (58)	he (54)	he (69)	on (63)	he (243)
	4^th^ (n)^a^	is (55)	she (38)	is (46)	he (61)	in (170)
	5^th^ (n)^a^	the (43)	in (35)	she (46)	with (51)	for (163)
	6^th^ (n)^a^	with (43)	with (34)	the (38)	in (49)	with (162)
	7^th^ (n)^a^	in (40)	on (33)	with (34)	for (43)	she (151)
	8^th^ (n)^a^	to (32)	for (31)	came (32)	she (42)	on (141)
	9^th^ (n)^a^	of (30)	to (29)	for (31)	the (37)	the (138)
	10^th^ (n)^a^	came (27)	came (24)	to (30)	to (33)	to (124)
Top 10 inside words in documents	1^st^ (n)^a^	he (57)	he (52)	he (63)	and (51)	he (220)
	2^nd^ (n)^a^	for (47)	she (35)	she (39)	he (48)	she (139)
	3^rd^ (n)^a^	and (26)	for (25)	and (34)	she(40)	and (131)
	4^th^ (n)^a^	bed (25)	dr (22)	bed (24)	for (27)	for (118)
	5^th^ (n)^a^	she (25)	and (20)	is (24)	dr (25)	dr (88)
	6^th^ (n)^a^	dr (23)	old (20)	to (23)	is (20)	to (84)
	7^th^ (n)^a^	to (22)	bed (19)	old (21)	on (20)	bed (80)
	8^th^ (n)^a^	the (21)	to (19)	yrs (21)	to (20)	is (76)
	9^th^ (n)^a^	her (18)	yrs (17)	all (20)	room (18)	old (72)
	10^th^ (n)^a^	old (18)	her (16)	for (19)	of (16)	all (61)

^a^ The notation “word, n” specifies that the word “word” occurred “n” times.

**Table 5:** Descriptive statistics of the 100 written documents produced by the RN.

Descriptor	Subdescriptor	Sample document	Patient type	Patient type	Patient type	Patient type	All
			Cardiovascular	Neurological	Renal	Respiratory	
Documents	Number of documents	1	25	25	25	25	101
	Number of words	167					8487
	Number of unique lemmas	92					1283
Number of words in a document	Minimum	167	26	37	35	32	26
	Maximum	167	181	170	239	120	239
	Mean	167	80.80	82.24	98.12	71.96	84.10
	SD	0	38.70	35.24	43.46	24.06	38.02
Number of unique lemmas in documents	Minimum	92	22	22	27	27	22
	Maximum	92	99	96	126	79	126
	Mean	92	53.64	54.48	63.84	48.60	55.50
	SD	0	19.83	17.44	21.84	12.80	19.35
Top 10 lemmas in documents	1^st^ (n)^a^	be (15)	be (115)	be (119)	be (126)	be (111)	
	2^nd^ (n)^a^	he (13)	and (95)	he (95)	and (100)	he (68)	
	3^rd^ (n)^a^	and (4)	he (75)	and (88)	he (79)	and (64)	
	4^th^ (n)^a^	to (4)	for (58)	she (63)	on (63)	she (57)	
	5^th^ (n)^a^	a (3)	she (44)	in (46)	she (59)	in (35)	
	6^th^ (n)^a^	headache (3)	the (43)	the (38)	with (51)	with (34)	
	7^th^ (n)^a^	it (3)	with (43)	have (36)	in (49)	on (33)	
	8^th^ (n)^a^	that (3)	in (40)	with (34)	for (43)	for (31)	
	9^th^ (n)^a^	the (3)	to (32)	come (33)	the (37)	to (29)	
	10^th^ (n)^a^	carotid (2)	of (30)	for (31)	to (33)	have (26)	
Number of highlighted text snippets in a document	Minimum						8
	Maximum						33
	Mean						16.15
	SD						5.29

^a^ The notation “word, n” specifies that the word “word” occurred “n” times.

### Evaluation Outcomes From Speech Recognition

**Figure 3:**
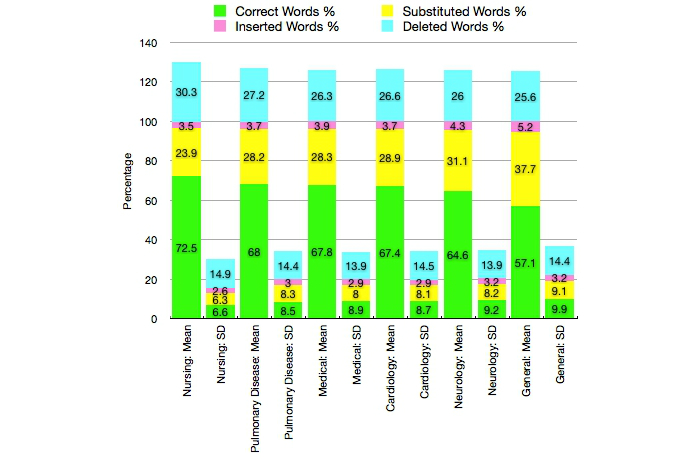
Speech recognition performance with the vocabularies of general, medical, nursing, cardiology, neurology, and pulmonary disease illustrated as a summary over the 100 documents. The notation of the x axis details the mean and SD for each Dragon vocabulary.

The best vocabulary for SR was *nursing*, resulting in the largest mean (5275/7277 words, ie, 0.725) and smallest SD (0.066) of correctly recognized words over the total of 7277 words (1 hour, 8 minutes, 5 seconds) in our 100 documents (Figure 3 shows this, see Multimedia Appendix 1). This correctness had the minimal, maximal, and median values of 0.547, 0.864, and 0.737 for this vocabulary. The *nursing* vocabulary also gave the largest number of correct words in 74 out of 100 cases. For the 25 cardiovascular patients, the matching vocabulary (ie, *cardiology*) gave more correct words than any other vocabulary only three times. For the 25 neurological patients with the *neurology* vocabulary and 25 respiratory patients with the *pulmonary disease* vocabulary, this number was four and zero, respectively. The number of times when the matching vocabulary gave more correct words than the *nursing* vocabulary was only four, three, and six for the cardiovascular, neurological, and respiratory patients, respectively. The *medical* vocabulary performed very differently from other vocabularies; its word distribution to correct, substituted, inserted, and deleted words had more inserted and deleted words, but less correct words. An example of speech-recognized text using the *nursing* vocabulary is given in Textbox 1.

When considering the different patient types and the *nursing* vocabulary, the mean of correctly recognized words was 0.733 for the 25 cardiovascular patients, 0.732 for the 25 neurological patients, 0.724 for the 25 renal patients, and 0.713 for the 25 respiratory patients with the respective SDs of 0.073, 0.059, 0.063, and 0.071. That is, SR was slightly easier on cardiovascular patients, on average. Also the minimal and maximal values for the word correctness (ie, 0.619 and 0.864) were the largest for this patient type.

In text relevant to the form, 836 unique errors were present when using the *nursing* vocabulary [63]. Substitutions and insertions were the most common error types. Nearly a fifth of word substitutions sounded exactly the same as the correct word and over a quarter of the substitutions had a PS percentage above 75. Half of the substitutions occurred with words shorter than 4 characters that were obviously harder for SR than longer words. The most common single-word substitutions were “years” versus “yrs” and “in” versus “and” (n≥20). This error type was generally related to proper names (a quarter of errors and some of them sounded exactly the same, eg, “Lane” vs “Laine”, and often were just spelling variants, for example, “Johnson” vs “Johnsson”) and singular versus plural forms (eg, “fibrosis” vs “fibroses”). In conclusion, around a quarter of substitutions were candidates for their correction, and most of these errors were not SR errors, but rather explained by our written documents. The most common insertions included short words (eg, “and”, “is”, “in”, “she”, “are”, “all”, “arm”, “for”, “the”, “he”, “that”, or “a”, *n*≥20), typically when the RN used “aa”, “mm”, “eh”, or other back-channels that were not included in the written free-form text documents. The majority of the insertion and deletion errors corresponded to functional words with little semantic meaning. The most common deletion was “is” (n=20). Almost all remaining errors were caused by the following four types of systematic differences between the written free-form text documents and SR: (1) Australian versus US spelling (eg, “ catheterisation” vs “catheterization”); (2) digits versus letters (eg, “0” vs “zero”); (3) the RN’s use of abbreviations and acronyms in her writing, but complete forms when speaking (eg, “AM” vs “this morning”, “obs” vs “observations”, and “K” vs “potassium”); and (4) RN’s typing mistakes (eg, “ arrythmia” vs “arrhythmia”).

**Speech-recognized text corresponding to the example record.**

TRANSCRIPTION OF SPOKEN, FREE-FORM TEXT DOCUMENT:

“On a bed three is Ken Harris, 71 years old under Dr Gregor. He came in with arrhythmia. He complained of chest pain this morning and ECG was and was reviewed by the team. He was given some anginine and morphine for the pain and he is still tachycardic and new meds have been ordered in the medchart. Still for pulse checks for one full minute. Still awaiting for echo this afternoon. His blood pressure is just normal though he is scoring MEWS of three for the tachycardia. Otherwise he still for monitoring.”

WRITTEN, FREE-FORM TEXT DOCUMENT:

“Ken harris, bed three, 71 yrs old under Dr Gregor, came in with arrhythmia. He complained of chest pain this am and ECG was done and was reviewed by the team. He was given some anginine and morphine for the pain. Still tachycardic and new meds have been ordered in the medchart. still for pulse checks for one full minute. Still awaiting echo this afternoon. His BP is just normal though he is scoring MEWS of 3 for the tachycardia. He is still for monitoring.”

WRITTEN, SPEECH-RECOGNIZED, FREE-FORM TEXT DOCUMENT USING THE NURSING VOCABULARY:

“Own now on bed 3 he is then Harry 70 is 71 years old under Dr Greco he came in with arrhythmia he complained of chest pain this morning in ECG was done and reviewed by the team he was given some and leaning in morphine for the pain in she is still tachycardic in new meds have been ordered in the bedtime is still 4 hours checks for one full minute are still waiting for echocardiogram this afternoon he is BP is just normal though he is scarring meals of 3 for the tachycardia larger otherwise he still for more new taurine.”

### Evaluation Outcomes From Information Extraction

**Figure 4:**
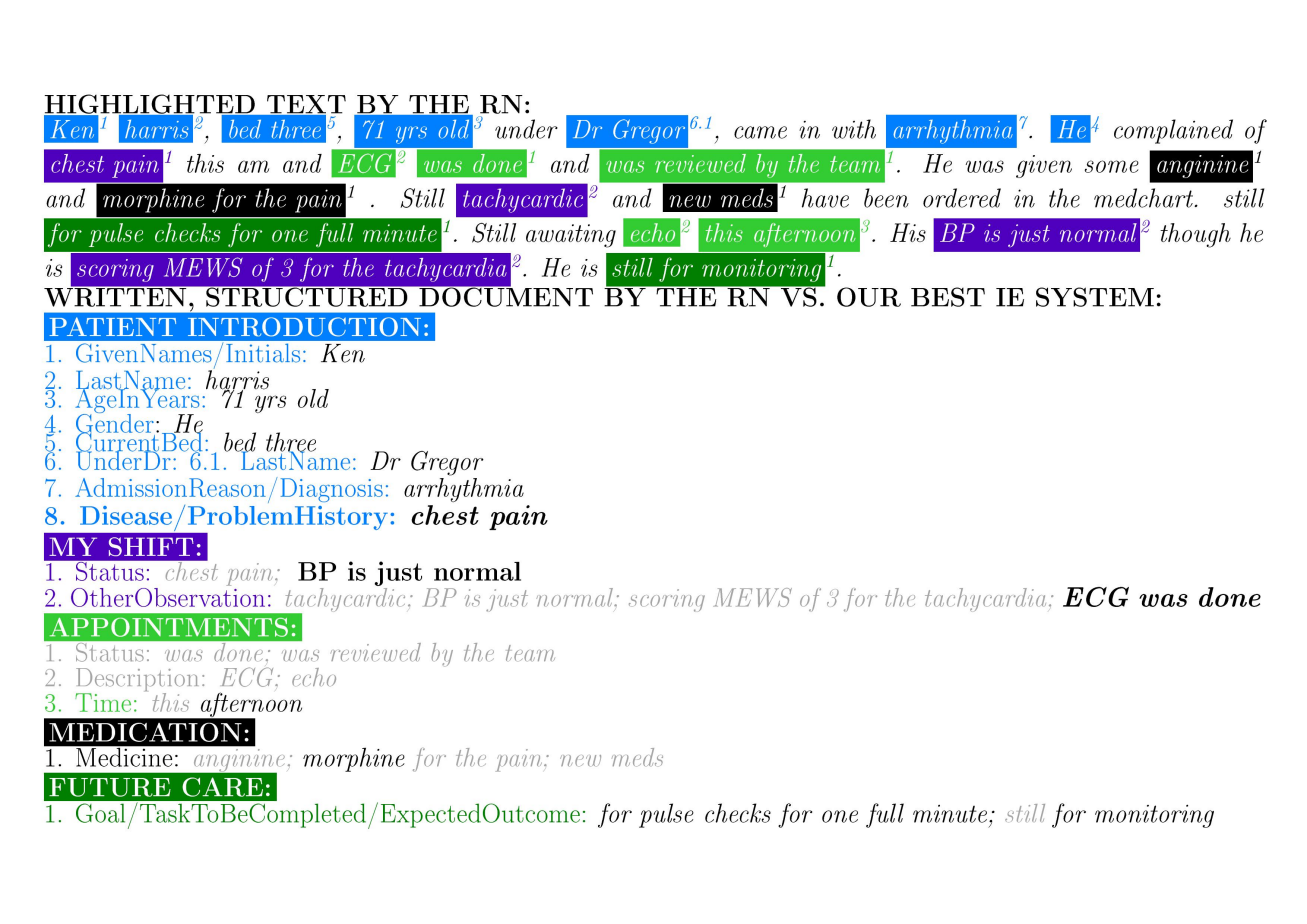
Automatically structured text that corresponds to our example document (Figure 1). When compared with the reference standard, added text is shown as bold and removed text is shown in grey. Risk-carrying errors include: (1) "chest pain" moving from "MY SHIFT, Status" to "PATIENT INTRODUCTION, Disease/problem history", (2) not identifying "tachycardic" and "scoring MEWS of 3 for the tachycardia" for "MY SHIFT, Other Observation", (3) not identifying "echo" for "APPOINTMENTS, Description", and (5) not identifying "anginine" and "new meds" for "MEDICATION, Medicine". RN: registered nurse and IE: information extraction.

**Figure 5:**
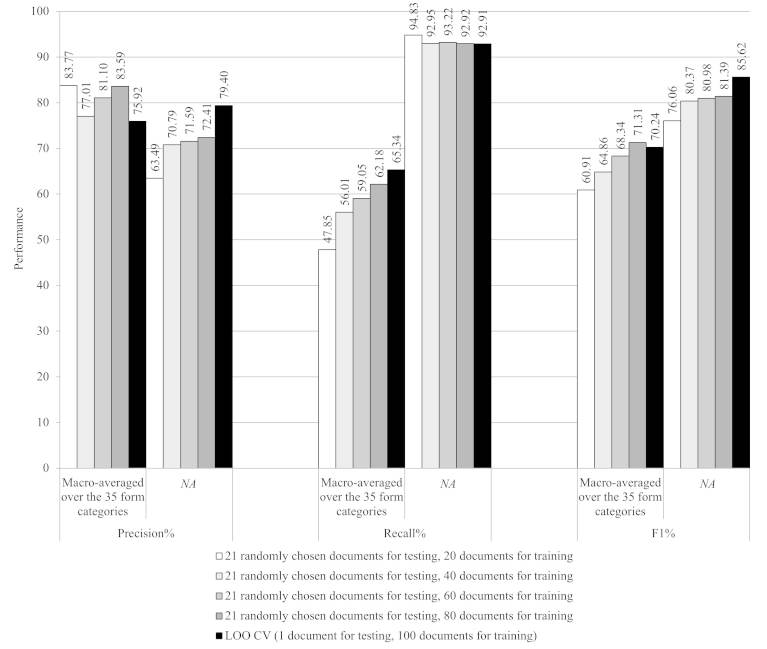
Learning curves for cross-validation settings that included training set sizes of 20, 40, 60, 80, and 100 (ie, leave-one-out) documents with mutually exclusive folds, which in combination covered all data. CV: cross validation; and LOO: leave one out.

Our best IE system classified 6349 out of the 8487 words correctly with respect to the 36 categories present in the RS (Figure 2). Figure 4 shows an example of an automatically structured document. The system performed excellently in filtering out irrelevant text (ie, *NA* category with 0.794 Precision, 0.929 Recall, and 0.856 F1 or 3481 correct out of 3771). The macro-averaged F1 over the 35 nonempty sub and subsubheading categories of the RS was 0.702 (Precision 0.759 and Recall 0.653). As expected, the larger amount of data for training, the better was the system performance (Figure 5 shows this). The system also performed substantially better in well-defined, compact categories (eg, perfect or nearly perfect Precision, Recall, and F1 in identifying the patient’s current room and bed, respectively) than in more abstract and verbose categories (eg, 0.217 and 0.496 F1 in identifying *other observations* for *MY SHIFT* and *goals, tasks to be completed,* and *expected outcomes* for *FUTURE CARE*, respectively).

**Figure 6:**
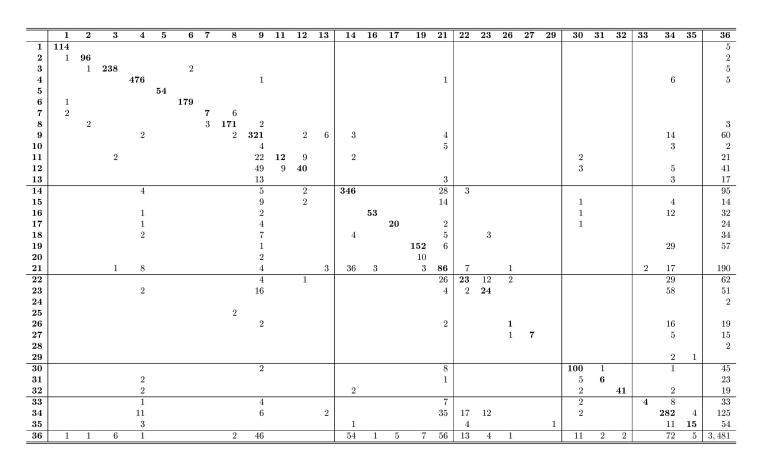
Confusion matrix between the reference standard (rows) and our best information extraction system (columns) in the 36-class multi-class classification task. Zero columns of 10, 15, 18, 20, 24, 25, and 28 have been removed for space constraints. For clarity, diagonal elements have been emphasized, and zero elements have been left empty. The category numbering corresponds to Figure 2. IE: information extraction.

Most frequent category confusions related to irrelevant words (Figure 6 shows 1057 false positives and 290 false negatives). Other common confusions included differentiating: (1) *APPOINTMENTS, Description*, *APPOINTMENTS, Status*, and *MY SHIFT, Activities of daily living* from *FUTURE CARE, Goal/task to be completed/expected outcome* (n=58, n=29, and n=29); (2) *Disease/problem history* and *Chronic condition* from *Admission reason/diagnosis* under *PATIENT INTRODUCTION* (n=49 and n=22); (3) *Other observation* from *Status* (n=36) and vice versa (n=28) under *MY SHIFT*; and (4) *FUTURE CARE, Goal/task to be completed/expected outcome* from *MY SHIFT, Other observation* (n=35), where the first category is always with respect to the RS and the second refers to our best IE system.

In comparison, the majority baseline achieved overall a very modest performance (macro-averaged Precision, Recall, and F1 of 0.051, 0.091, and 0.065 over the 35 form categories and zero Precision, Recall, and F1 for *NA*). Its Precision, Recall, and F1 in the majority category were 1.00, 0.051, and 0.093. The random baseline was even weaker (macro-averaged Precision, Recall, and F1 of 0.015, 0.026, and 0.019 over the 35 form categories and 0.372, 0.030, and 0.055 for *NA*).

Each system feature contributed to the 36 categories differently (see Multimedia Appendix 1). However, on “average” (*μ*) over the 36 categories, Lemma was the most influential type (*μ* = 1**.**07), followed by Top 5 candidates (*μ* = 0**.**69), Part of speech (POS*, μ* = 0**.**56), Top mapping (*μ* = 0**.**35), and named entity recognition (NER, *μ* = 0**.**26). If considering this decrease in the macro-averaged F1 over the 35 form categories, the five types that influenced the most were Location (0.89), Top 5 candidates (0.25), POS (0.24), Basic governors (0.23), and Parse tree (0.16). In filtering out irrelevant words, they were POS, Lemma, Basic dependents, Location, and Top 5 candidates, with the decrease in the F1 of 0.0151, 0.0060, 0.0050, 0.0034, and 0.0023 respectively. This demonstrates that both the syntax and semantics together with the word location in the document is advantageous.

In the highest-level classification task with all but the *MY SHIFT* category present in the RS, the system trained on the highest-level annotations outperformed the system trained on the subheading and subsubheading level annotations (6731 vs 6710 words out of the 8487 words right, Figure 2). The respective category-specific statistics were: the F1 of 0.919 versus 0.918 for *PATIENT INTRODUCTION* (1882 vs 1880 correct out of 2064); the F1 of 0.737 versus 0.712 for *MY SHIFT* (915 vs 926 correct out of 1353); the F1 of 0.263 versus 0.279 for *APPOINTMENTS* (101 vs 109 correct out of 393); the F1 of 0.624 versus 0.650 for *MEDICATION* (153 vs 159 correct out of 262); the F1 of 0.547 versus 0.536 for *FUTURE CARE* (328 vs 320 correct out of 644); and the F1 of 0.863 versus 0.867 for *NA* (3352 vs 3316 correct out of 3771).

### The National Information and Communications Technology, Australia Speech to Clinical Text Demonstration System

**Figure 7:**
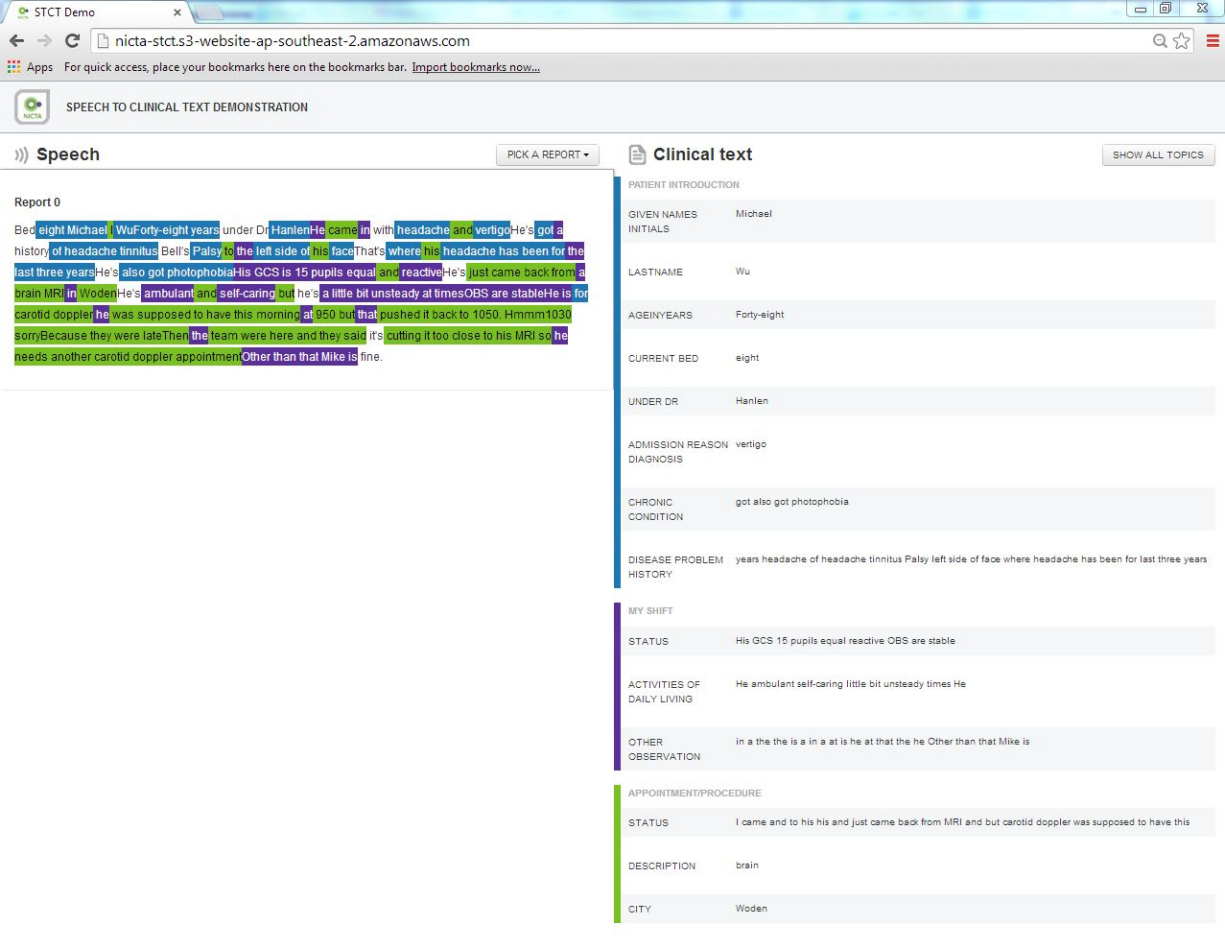
National Information and Communications Technology, Australia (NICTA) speech to clinical text demonstration system that visualizes the example record.

To demonstrate the SR and IE system design and workflow, we implemented a Web-app, written in the HyperText Markup Language, version 5 to allow any Web-browser to use it (Figure 7 show this) [64]. In particular, this means that the app is iPad compatible.

As an input, the app receives a form structure and an XML document, which includes all information needed to fill out this form. That is, the input has typed or speech-recognized text documents and their word-by-word classification with respect to the form categories.

The user (eg, a nurse) can choose a report to be structured from the *“*Pick a report” menu, see this written, free-form text on the left-hand side, and the filled-out form is given on the right-hand side. The report text is highlighted with respect to the headings of the form. In this way, the full text context never gets lost. The user can choose to see either the entire form (ie, “Show all topics”) or only the subheadings and subsubheadings with extracted content (ie, “Only show available topics”).

Extending the app to other IE tasks is straightforward by simply updating the input. However, we need to emphasize that this app performs visualization and not processing. That is, the spoken documents need to be converted to writing (by typing or SR) and classified with respect to the form structure (by manual highlighting or automated IE) in advance.

SR has not been included in the app. This is mainly because of the licensing constraints related to using a domain-specialized SR method (for a Microsoft Windows computer) that also needs to be trained to each speaker individually. However, also the aspect of being able to demonstrate the app in a noisy conference, technology festival, and other showcase environments led us to not include SR in the app.

##  Discussion

### Principal Results

Cascaded SR and IE to fill out a handover form for clinical proofing and sign-off provide a way to make clinical documentation more effective and efficient. This way also improves accessibility and availability of existing documents in clinical judgment, situational-awareness, and decision making. Thereby, it contributes to the health care quality and people’s health.

This cascading also evokes fruitful research challenges. First, conducting SR at clinical wards with noisy background and accented speakers is much more difficult than in a peaceful office. Second, its errors multiply when cascaded with IE. Third, every system error may have severe implications in clinical decision making. However, neither shared evaluation sets, nor baseline methods exist for this task.

In this paper, we have opened realistic, but synthetic data, methods, and evaluations related to clinical handover, SR, and IE to the research community in order to stimulate research and track continuous performance improvements in time. We have also introduced a Web app to demonstrate the system design and workflow.

### Limitations

#### Setting for the Study

A real hospital setting cannot be idealized or modeled in a laboratory. Although we have attempted to capture the main components of a nursing handover scenario, there are several limitations in the data.

These limitations represent opportunities for future data gathering exercises. First, we used a single narrative voice rather than a team environment. In order to further develop any real system, collection of multiple voices communicating in a group setting is needed. Second, we did not include patient responses. In the recorded data from real nursing scenarios, patients rarely contributed to the conversation. Third, the data comprises 100 full verbatim documents. This provides a low power to any statistical analysis, and hence more data are always beneficial.

#### Performance Evaluation and Error Analysis

A detailed performance evaluation and error analysis of the system as a whole (ie, extrinsic evaluation) and each of its components (ie, intrinsic evaluation) is a crucial step in the development of cascaded pipeline apps [65,66]. At their best, SR can be only a percentage from perfect, and according to our findings, only a quarter of substitution errors could be considered as correction candidates. Similarly with our IE component, the category-specific performance is at its best perfect, and altogether three-fourths (6349) of all 8487 words are correctly classified by our best system. The system performance is also convincing in filtering out irrelevant text (ie, 0.86 F1).

These rates of sound-alike SR-errors and slightly incorrect highlighting boundaries are not likely to harm a document’s human readability. This is because the context around the highlighted text snippets is likely to assist in reading the text correctly. However, the extrinsic performance of this cascaded system remains to be formally evaluated.

Every corrected error is one less potential error in clinical decision making and in SR, a substantial amount of errors occur with words that are phonetically similar to each other. Based on our error analysis, the correction method should consider the following five characteristics: (1) PS between words or word sequences; (2) detection and correction of errors in proper names, by using, for example, other parts of a given patient’s record; (3) difference between single-word and multi-word errors; (4) proofing for spelling and grammar; and (5) clear marking of automatically corrected words and possibility to choose a correction candidate interactively from a ranked list.

### Comparison With Prior Work

Clinical SR has resulted in 1.3-5.7 times faster turnover-time in scientific studies [62]. The impact of SR on documentation time has been studied at two US emergency departments with a report turnover-time of less than 4 minutes, and proofing-time of 3 minutes, 39 seconds [67]. For transcription by hand, the respective times are nearly 40 minutes, and 3 minutes, 46 seconds. Similar conclusions on freeing up time have been published from three US military medical teaching facilities (ie, 19 hours vs 89 hours) [68], over forty US radiology practices (ie, 16 hours vs 48 hours) [69], a Finnish radiology department (ie, 12 hours vs 25 hours) [70], and 5011 US surgical pathology reports (ie, 72 hours vs 96 hours) [71]. When comparing the clinical workflows of SR to transcription by hand followed by proofing and sign off, the capability to use SR produces nearly two-thirds of the signed-off reports in less than an hour at the aforementioned Finnish radiology department, while this proportion is a third for transcription by hand [70]. This efficiency gain is evident also in the aforementioned longitudinal study on 5011 US surgical pathology reports [71], SR with proofing by hand increases the proportion of the reports signed off in less than a day from a fifth for time before SR, through a quarter during the first 35 months of SR use, to over a third after this initialization period. The respective proportions for the reports signed off in less than two days are over half, nearly two-thirds, and over two-thirds.

Clinical SR achieves an impressive word correctness percentage of 90-99, with only 30 to 60 minutes of training to a given clinician’s speech. In other words, correcting SR errors by hand as a part of proofing is not likely to be time consuming. This recognition rate is supported by studies using the speech of twelve US-English male physicians on two medical progress notes, one assessment summary, and one discharge summary [72]; two US-English physicians’ speech on 47 emergency-department charts [67]; and the speech of seven Canadian-English pathologists, and one foreign-accented researcher on 206 surgical pathology reports [73]. In our previous study [36] that uses the speech of two Australian-English female nurses and one Australian-English male physician on six nursing handover documents, the correctness is up to 0.79, while now it was 0.73. Differences in the correctness across different systems are negligible (ie, 0.91-0.93 for IBM ViaVoice 98*,* General Medicine; 0.85-0.87 for L&H Voice Xpress for Medicine 1.2, General Medicine; and 0.85-0.86 for Dragon Medical 3.0) [72]. In comparison, the report-wise error rate in word correctness is 0.4 for transcribing clinical text by hand and 6.7 for SR [73].

Similarly to the good correctness of clinical SR, clinical IE has gradually improved to exceed F1 of 0.90 in 1995-2008 [10]. It is most commonly used for content extraction, structuring, and enrichment to support diagnosis coding, decision making, and surveillance in health care. Other typical applications are deidentifying records for research purposes and managing clinical terminologies. This processing focuses on processing chest and other types of radiography reports, discharge summaries, echocardiogram reports, and pathology reports. However, the 170 reviewed studies do not address handover. Our performance is comparable to this; when considering the 50 mutually exclusive categories in IE, our performance is 0.86 for irrelevant text and up to perfect (ie, 1.00) for the remaining 35 nonempty form categories. Our performance is superior to our previous study [36] on 150 Australian handover documents and five main headings, F1 is slightly (ie, +0.01) better now, while the macro-averaged F1 for the form categories is the same.

The benefits of the combined use of SR and IE for handover documentation are twofold [36]. First, this approach stores all information along the workflow of having the verbal handover, using SR in real time to transcribe the recording, storing the content also as an audio recording, using IE in real time to fill out the handover form from the transcription for proofing, tracking the proofing changes, and signing off the document. In this way, clinicians can always keep the context of information, track changes, and perform searches on both the transcriptions and forms. The editing history can also be used to improve SR and IE correctness. Second, the approach makes the record drafts available and accessible almost instantly to everyone with an authorized access to a particular patient’s documents. The speech-recognized transcription for a minute of verbal handover (approximately 160 words) is available only 20 seconds after finishing the handover with real time SR. Automated structuring through IE is almost instant and avoids problems related to subjectivity when structuring by hand. In comparison, clinicians would need to wait for almost four minutes for the hand-written transcription if they had a ward clerk to write the notes as they speak. This approach of using a clerk, either in real time or later on by the end of the shift, is also more prone to errors than clinicians, supported by a SR and IE system, writing the notes themselves in real time; if interpolating from the rate of information loss percentage from 60 to 100 after 3-5 shifts if notes are taken by hand, or not taken at all [4,74], more than an eighth of the information gets lost during one shift.

## Multimedia Appendix 1

Additional illustrations and guidelines for creating showcase data.

## Abbreviations

ANA: American Nurses Association
CLEF: Conference and Labs of the Evaluation Forum
CRF: conditional random field
CV: cross validation
IE: information extraction
LOO: leave one out
NA: not applicable
NER: named entity recognition
NICTA: National Information and Communications Technology, Australia
NLP: natural language processing
POS: part of speech
PS: phonetic similarity
RN: registered nurse
RS: reference standard
SR: speech recognition
WAV: WAVeform (audio format)
WMA: Windows Media Audio
