# Supplementary material for: Benchmarking Clinical Speech Recognition and Information Extraction: New Data, Methods, and Evaluations
Source: JMIR Med Inform. 2015 Apr 27;3(2):e19. doi: 10.2196/medinform.4321 (PMC4427705; doi:10.2196/medinform.4321)

Multimedia Appendix

Additional Illustrations

For Introduction

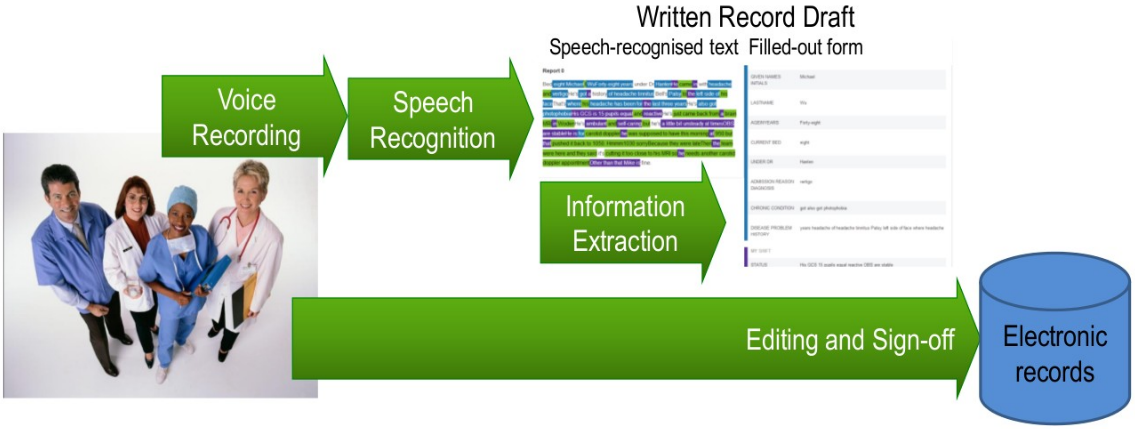

A processing pipeline that transforms verbal clinical handover information into electronic structured records automatically.

For Methods

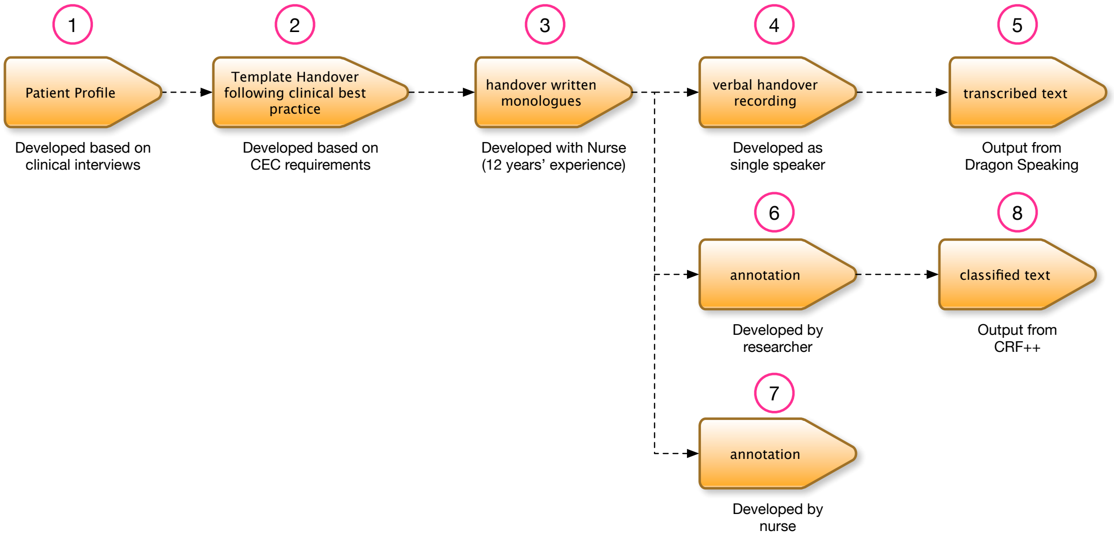

A workflow architecture that consisted of eight steps. Patient profiles (summaries) were created based on experience and clinical interviews (1). A template handover outline was created (2) matching best practice; A handover event, comprising the text of a nurse speaking to a virtual colleague was created through a creating writing exercise (3) and recorded using a personal microphone (4) to simulate inward acoustics. The audio file was passed through a commercial speech-to-text analysis engine (5) to evaluate SR errors. Separately, the original text (3) was annotated by a practicing nurse (6) to highlight the components of the text that matched the hospital pro-forma and re-annotated by an informatics researcher (7) to evaluated inter-annotator disagreement. The annotated text (6) was used to train an open-source classifier (8) to automatically generate pre-filled handover forms.

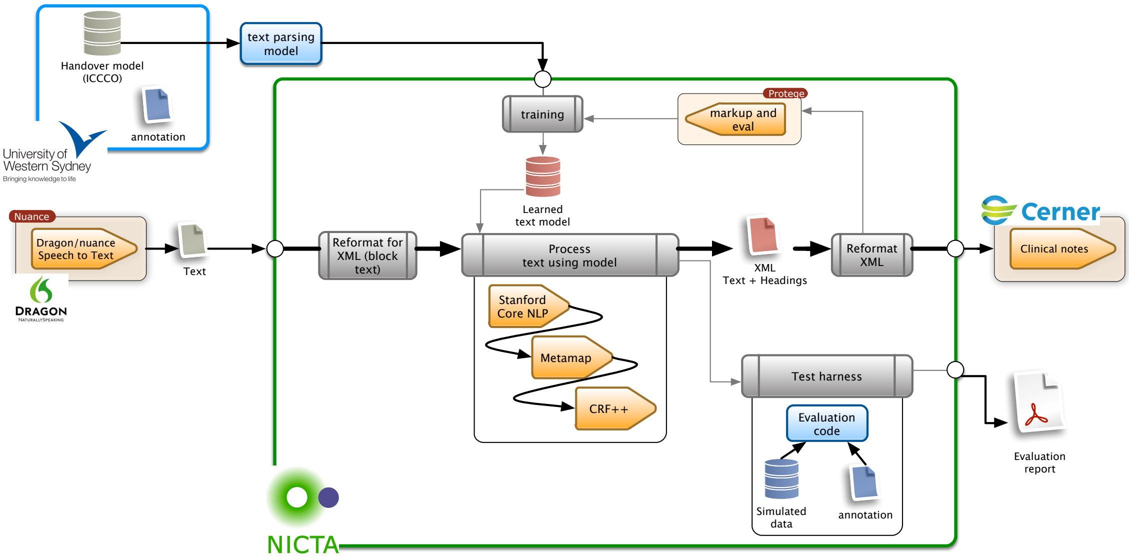

Application of clinical dataset for classified pre-filled nursing handover documentation. Corporate references describe its interconnections in a health information system. The entire section denoted by the green rectangle will be provided as open source software and the data described here may be used for training and evaluation of the system.

**HANDOVER NURSE**		
Title		
Given names/ initials		
Last name		
Allocated rooms		
Allocated beds		
**PATIENT INTRODUCTION**		
Title		
Given names/ initials		
Last name		
IDnumber		
Date of birth	DOB year	
	DOB month	
	DOB day	
Age in years		
Gender		
Current room		
Current bed		
Under Dr	Given names/ initials	
	Last name	
Admission reason/ diagnosis		
Allergy		
Chronic condition		
Disease/ problem history		
Care plan		
**MY SHIFT**		
Status		
Contraption		
Input/ diet		
Output/ diuresis/ bowel movement		
Wounds/ skin		
Activities of daily living		
Risk management		
Other observation		
**APPOINTMENTS**		
Status		
Description		
Clinician	Title	
	Given names/ initials	
	Last name	
Date and time	Year	
	Month	
	Day	
	Time	
Place	City	
	Hospital	
	Ward	
**MEDICATION**		
Medicine		
Dosage		
Status		
**FUTURE CARE**		
Alert/ warning/ abnormal result		
Goal/ task to be completed/ expected outcome		
Discharge/ transfer plan		

The handover form that was developed for this study.

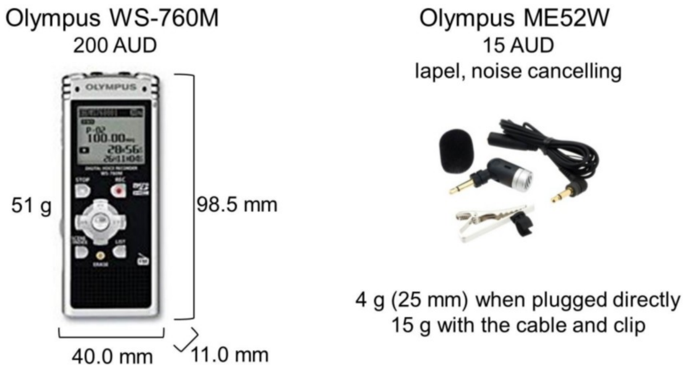

The recorder and microphone that were used in our data collection.

For Results

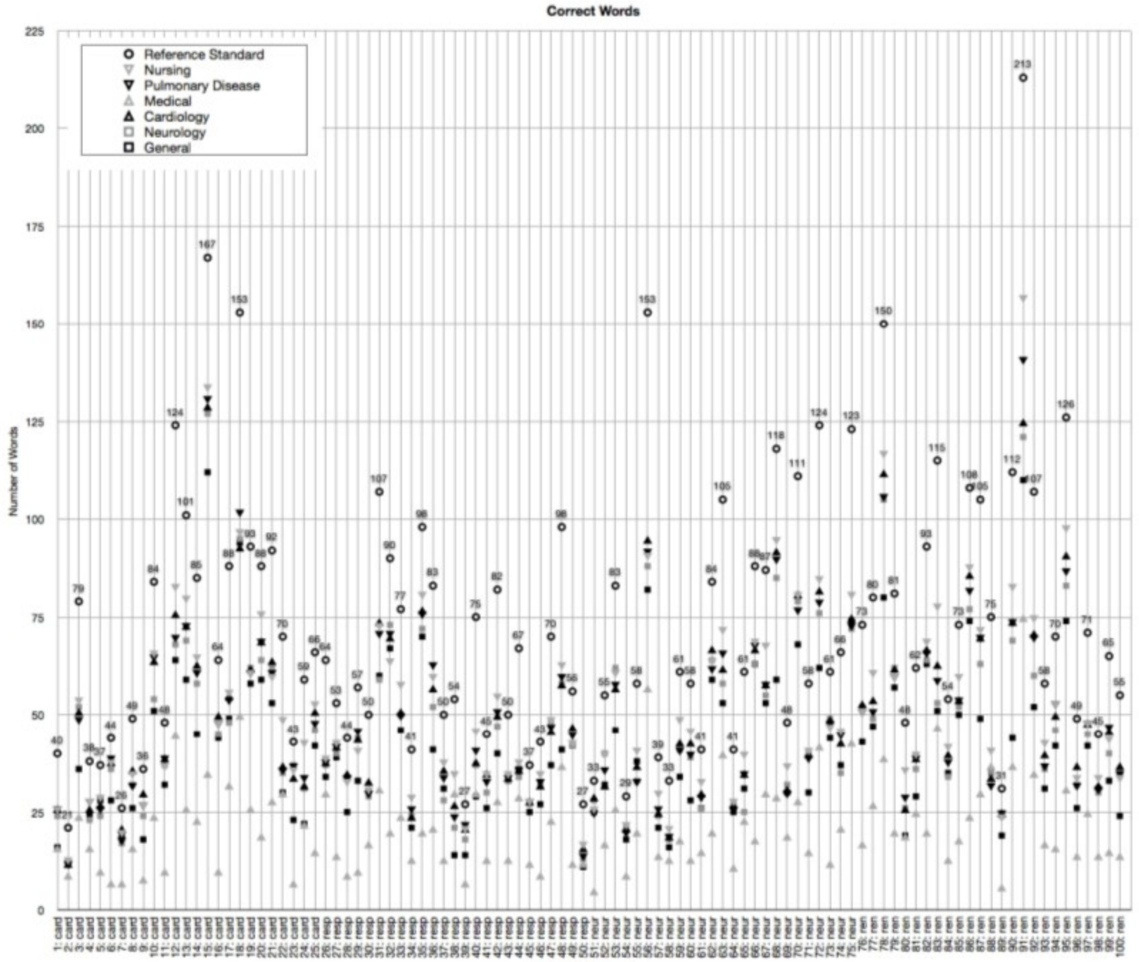

The suitability of different vocabularies, as defined by the number of correctly recognized words, illustrated separately for each document.

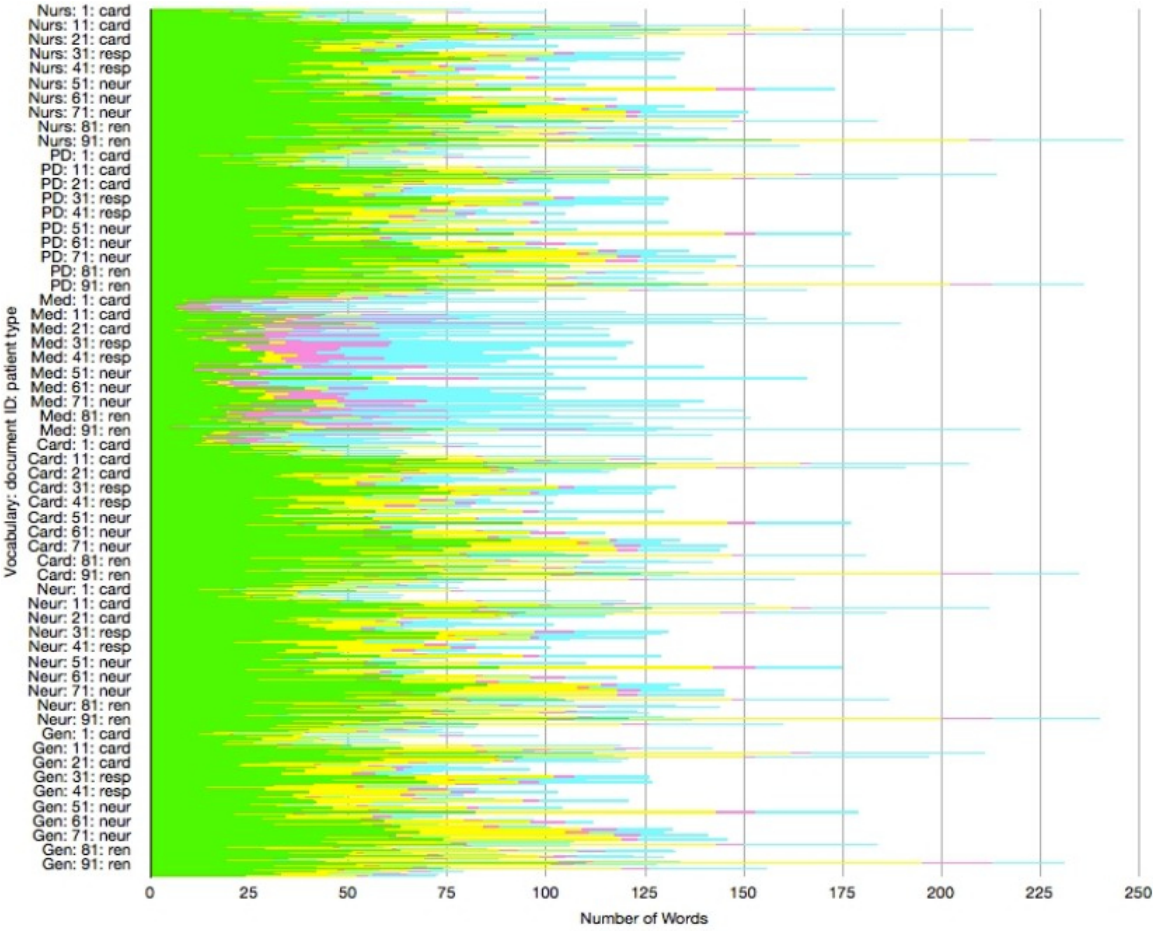

Speech recognition performance with the vocabularies of general (Gen), medical (Med), nursing (Nurs), cardiology (Card), neurology (Neur), and pulmonary disease (PD) illustrated separately for each document.

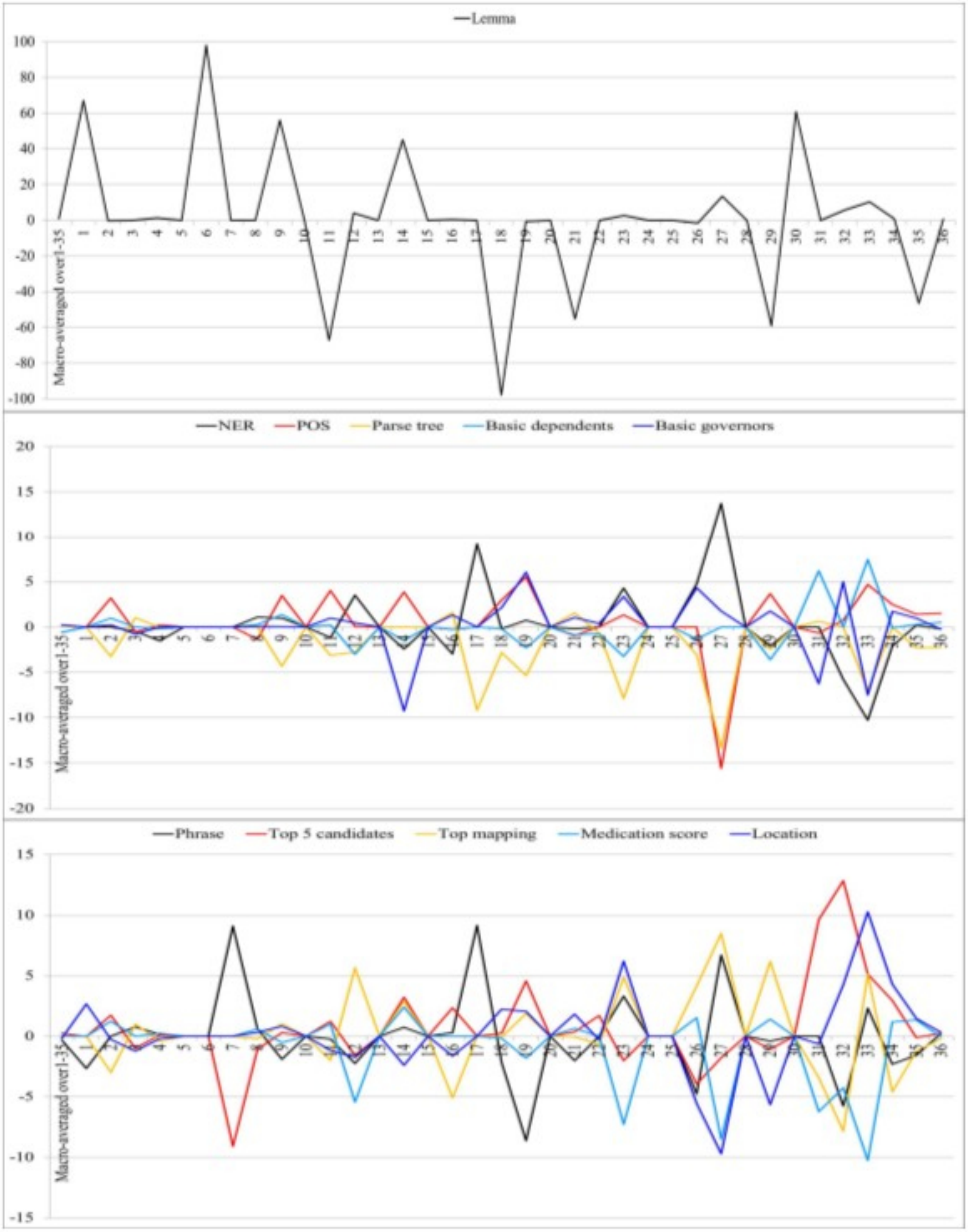

Contribution of each feature type to our best system. We measured the decrease in the F1 percentage between the system with all feature types (i.e., the best system) and a system without each feature type in turn is measured using LOO CV. The category numbering corresponds to Figure 2 of the Paper.

Speech-to-Text and Information Extraction for Nursing Records

Guidelines for Creating Showcase Data

Author: Hanna Suominen, NICTA
Date: 30 Jan 2013

Introduction

Clinical handover or handoff is defined as the transfer of professional responsibility and accountability for some or all aspects of care for a patient or group of patients, to another person or professional group on a temporary or permanent basis. It can take place, for example, at nursing shift change.

Clinical handover is a procedure used universally by nurses to promote care continuum. Misunderstandings, breakdowns, and other failures in this communication happen. They can lead to severe consequences, including, preventable sentinel and adverse events. Verbal clinical handover typically provides an accurate representation of a given patient's background and current state of clinical management. However, over two thirds of this valuable verbal information is lost after five consecutive shifts if handover notes are taken by hand. If no notes are taken, all verbal information is lost after three shifts.

NICTA is developing technology to improve clinical handover communication by taking handover notes semi-automatically. First, verbal clinical handover is converted to a free-text document using SR. Second, a structured form is filled with text snippets extracted from the free-text document using IE. This automation provides a systematic approach of incorporating a document draft to computerized medical records systems after its manual clinical review and editing.

Task 1: Creating Free-Text Documents

The first task consists of creating 100 documents describing realistic but imaginary verbal handovers. Please imagine that you are working as yourself in a medical ward and are delivering verbal handovers to another nurse at nursing shift change by your patient's bedside. Please assume that you are NOT discussing with another nurse but rather having a monologue that includes all handover information at once. Please write a text document (a txt file) that captures this handover one file per one patient and all text as one paragraph. Please write as if you were talking and use words and phrases that you would normally use. Each document should be from 100 to 300 words. Please spend 5-15 minutes to imagine and type each scenario. An example document is shown below as well as some further requirements for the patient cases.

We want 100 unique patients (i.e., only one document per patient). Some patients should have been just admitted to the ward, some should have been there for some days already and some should be almost ready to be discharged. Some patient should have a short inpatient period and some longer. Reasons for admission should be acute conditions, but some patients should have chronic diseases too. To create a balanced dataset, we wish to have only adult patients (but both young and old), including respiratory, cardiovascular, renal, and neurological conditions, 25 patients per each condition category.

You can assume that both you and the other nurse know some patients quite well already whilst you and/or the other nurse are not so familiar with other patient cases. Please use navigate to the folder Profiles/100profiles to see the pre-set profiles of the 100 cases (1.doc, 2.doc, 3.doc, … , 100.doc). After familiarizing yourself with a profile, please open the respective txt file (1.txt, 2.txt, 3.txt, … , 100.txt) and write your 100-300-word document there. Please remember to save your txt files.

Example document: *Bed eight, Michael I Wu. Forty-eight years under Dr Hanlen. He came in with headache and vertigo. He's got a history of headache, tinnitus, Bell's Palsy to the left side of his face. That's where his headache has been for the last three years. He's also got photophobia. His GCS is 15 pupils equal and reactive. He's just came back from a brain MRI in Woden. He's ambulant and self-caring but he's a little bit unsteady at times. OBS are stable. He is for carotid doppler, he was supposed to have this morning at 950 but that pushed it back to 1050. Hmmm. 1030, sorry. Because they were late. Then the team were here and they said it's cutting it too close to his MRI so he needs another carotid doppler appointment. Other than that Mike is fine.*

End-result: 1.txt, 2.txt, 3.txt, … , 100.txt

Estimated time: 5-15 min per document. 8-25 h in total, preferably around 15 h

Task 2: Marking the Text Snippets for Form Filling

An example form is given below. Note that not all documents will include all information specified in the form (i.e., some slots will not be filled in with text snippets). Further, some documents have relevant information to a given heading multiple times (e.g., the patient's given name of Michael and Mike or two care team members).

Please use Protégé Knowtator as guided and practiced in our training session to mark the snippets of text relevant to each subheading (see an example highlighting below). The program has been installed on your computer. Please proceed a document by document until you have marked all classes for all documents.

End-result: annotated.pins, annotated.pons, and annotated.pprj files that capture all markings (all subheadings for all documents)

Estimated time: 15-30 min per document. 25-50 h in total, preferably around 35 h

Task 3: Creating Speech-Recognized Free-Text Records

In this task, you will speak all 100 documents of the task 1 out loud as naturally as you can at NICTA. First, we will record what you said using a digital voice recorder and microphone in order to be able to repeat the experiments in the future. The use of the recorder and microphone will be practiced before the actual recording and you can always record a document again if you are not happy with the way you sound. Second, when you are happy with all your document recordings, we will initialize SR software together with you and then use it to convert your audio files to text.

End-result: 1.wma, 2.wma, 3.wma, … , 100.wma and the respective Dragon outputs

Estimated time: 5-10 min per document. 8-16 h in total, preferably around 12 h

Example form:

Handover Nurse

Title	RN
Given names/initials	L Jamie
Last name	Hanlen
Allocated rooms	2
Allocated beds	1-8

Patient Introduction

Title		
Given names/initials		
Last name		
IDnumber	medical record number 183664830183883	
Date of birth	DOB year	1981
	DOB month	11
	DOB day	11
Age in years	31	
Gender	He	
Current room	3	
Current bed	1	
Under Dr	Given names/initials	
	Last name	
Admission reason/diagnosis	came in with SOB	
Allergy	penicillin	
Chronic condition	DM type 2	
Disease/problem history		
Care plan		

My Shift

Status	
Contraption	
Input/diet	
Output/diuresis/bowel movement	
Wounds/skin	
Activities of daily living	
Risk management	
Other observation	

Appointment/procedure

Status	planned	
Description	MRI scan	
Clinician	Title	
	Given names/initials	
	Last name	
Date and time	Year	
	Month	
	Day	
	Time	
Place	City	
	Hospital	
	Ward	

Medication

Medicine	sliding scale insulin
Dosage	
Status	started 5 years ago

Future Care

Alert/warning/abnormal result	may be MRSA+
Goal/task to be completed/expected outcome	
Discharge/transfer plan	

Example highlighting:

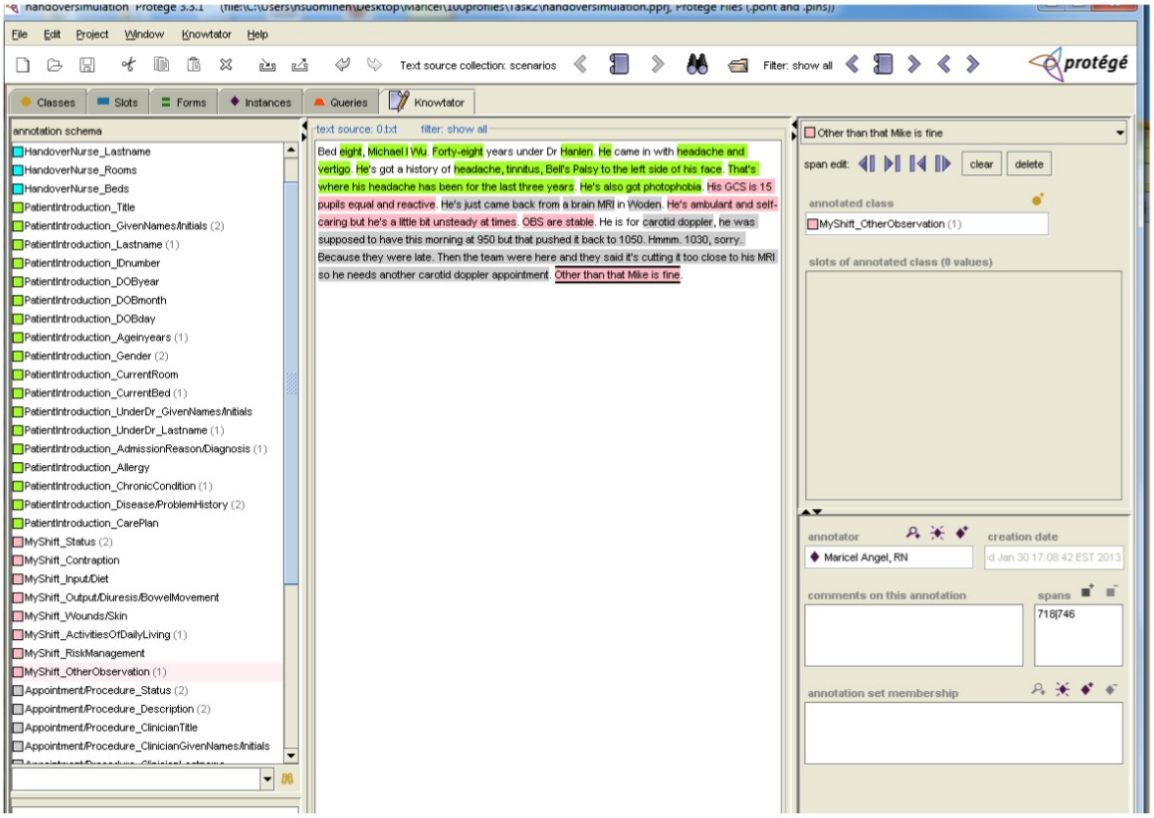

Supplement: Supplementary file 1 [file medinform_v3i2e19_app1.pdf]
